# Cellulose-Based Hydrogels for Wastewater Treatment: A Concise Review

**DOI:** 10.3390/gels7010030

**Published:** 2021-03-18

**Authors:** Maimuna Akter, Maitry Bhattacharjee, Avik Kumar Dhar, Fahim Bin Abdur Rahman, Siddika Haque, Taslim Ur Rashid, S M Fijul Kabir

**Affiliations:** 1 Department of Environmental Management, Independent University Bangladesh, Dhaka 1229, Bangladesh; maimuna_041@yahoo.com (M.A.); frahman@email.sc.edu (F.B.A.R.); 2 Department of Textile Engineering, Shyamoli Textile Engineering College, University of Dhaka, Dhaka 1207, Bangladesh; maitry1992moni@gmail.com (M.B.); avikkumardhar1990@gmail.com (A.K.D.); 3 Department of Chemical Engineering, University of South Carolina, Columbia, SC 29208, USA; 4 Faculty of Textile Engineering, BGMEA University of Fashion and Technology, Dhaka 1230, Bangladesh; siddika@buft.edu.bd; 5 Wislon College of Textiles, North Carolina State University, Raleigh, NC 27606, USA; turashid@ncsu.edu

**Keywords:** wastewater, heavy metal, dye, hydrogel, cellulose

## Abstract

Finding affordable and environment-friendly options to decontaminate wastewater generated with heavy metals and dyes to prevent the depletion of accessible freshwater resources is one of the indispensable challenges of the 21st century. Adsorption is yet to be the most effective and low-cost wastewater treatment method used for the removal of pollutants from wastewater, while naturally derived adsorbent materials have garnered tremendous attention. One promising example of such adsorbents is hydrogels (HGs), which constitute a three-dimensional polymeric network of hydrophilic groups that is highly capable of adsorbing a large quantity of metal ions and dyes from wastewater. Although HGs can also be prepared from synthetic polymers, natural polymers have improved environmental benignity. Recently, cellulose-based hydrogels (CBHs) have been extensively studied owing to their high abundance, biodegradability, non-toxicity, and excellent adsorption capacity. This review emphasizes different CBH adsorbents in the context of dyes and heavy metals removal from wastewater following diverse synthesis techniques and adsorption mechanisms. This study also summarizes various process parameters necessary to optimize adsorption capacity followed by future research directions.

## 1. Introduction

Water is the most abundant natural resource on the planet; however, only about 3% of current water reserves are freshwater, while less than one-third of this freshwater is usable for different household, agricultural, and industrial activities [1,2,3]. While water demand enormously increases, the availability of freshwater is being exhausted because of an escalation in pollution, thus causing water scarcity for modern civilization [4,5]. Rapid industrialization, haphazard urbanization, and various anthropogenic activities along with improper waste disposal direct to such an increase in wastewater. Dyes and heavy metals are the two most typical contaminants found in industrial wastewater, which catastrophically affects sustainable ecosystems [6,7,8,9]. The existence of dyes, even at low concentrations, limits sunlight penetration into water that causes a significant reduction of dissolved oxygen, creating critical health risks to the aquatic living bodies. In many cases, dyes induce anaerobic digestion to produce different carcinogenic compounds, which can enter the food chain via aquatic organisms [10]. However, the amount of dye disposal into different water sources is still significant. For instance, over 7 × 10^5^ tons of different reactive dyes are produced annually, whereas about 5–10% of these dyes end up in the industrial effluents [11]. Heavy metals, on the other hand, cause serious threats to human health because of their high carcinogenic and toxic nature. For example, a chronic intake of arsenic (As) can cause severe diseases such as kidney, prostate, bladder, or liver cancer. Chromium (Cr) is another highly toxic metal that unpleasantly affects human bodies as well as various aquatic species [12]. The hazardous nature of both dyes and heavy metals to public health and the natural ecosystem highlights the necessity of efficient treatment of industrial wastewater. Table 1 summarizes the adverse impacts of heavy metals and synthetic dyes on human health and the environment.

Adsorption is the most common and effective method used for wastewater treatment because it is convenient, simple, less expensive, and has no harmful by-products [37,38,39,40]. Activated carbon as an adsorbent is highly efficient that is often used commercially to remove pollutants from wastewater. However, the excessive cost and complicated regeneration process limit the widespread utilization of activated carbon for wastewater treatment [41]. Although recent studies have discovered numerous adsorbents for the removal of dyes and heavy metals from industrial effluent, the use of these adsorbents for bulk treatment of wastewater is still challenging [42,43]. HGs are three-dimensional polymer networks, have attracted incredible attention nowadays to eliminate contaminants from waste streams due to their high removal efficiency [44,45,46,47]. HGs are highly porous and comprised of numerous hydrophilic functional groups (e.g., –OH, −COOH, −NH_2_, −SO_3_H, −CONH_2_, etc.) that enable the adsorption and retention of a large volume of water during the treatment process and eventually cause up to the complete removal and recovery of aqueous dyes and heavy metals [48,49]. However, most of the existing HGs are derived from petrochemicals, which are neither renewable nor biodegradable. CBHs derived from cellulose, the most abundant polymer in nature, are superabsorbent, durable, biodegradable, biocompatible, and non-toxic [50]. Recently, researchers have reported almost complete removal of dyes and heavy metals from wastewater using different CBHs. For instance, Zhou et al. [51] achieved ≈90% of Pb^+2^ removal efficiency (adsorption capacity of 171 mg/g) within four hours of the adsorption process using carboxylated cellulose nanofibril-based hydrogels. Such high adsorption values were also obtained for Cu^2+^ (182–230 mg/g), Ni^2+^ (200 mg/g), and Hg^2+^ (140 mg/g) using different types of CBHs [51,52,53,54] Recently, Deng et al. [55] successfully removed almost 100% Congo Red dyes with 166.1 mg/g saturation adsorption using chitosan and cellulose. Numerous scopes of using CBHs still exist, since the modification of HG functional groups enhances its adsorption effectiveness. 

While numerous research works have been published using CBHs to treat wastewater streams, the lack of review in this specific area is the motivation of the present work. Table 2 demonstrates the gap in the literature based on the recently published review articles that are closely aligned to the proposed topic. A recently published book chapter has covered a few parts of the topic; however, it lacks the critical content, such as different adsorption mechanisms required to develop a comprehensive understanding of the topic [56]. This review is systematically designed to include critical information on the topic such as the development of CBHs for the dye and heavy metal adsorption from industrial effluent, adsorption mechanism, and factors affecting the adsorption capacity along with future outlook.

## 2. Preparation of CBH

A plethora of challenges generated by the excessive use of fossil resources and non-biodegradable materials result in shifting the attention of researchers toward renewable and environmentally safe materials. At this juncture, biopolymers are used in different areas such as agriculture, food packaging, biomedical applications, and wastewater treatment [55,64,65,66,67,68,69,70]. Similarly, when it comes to the removal of dyes and heavy metals by adsorption process, biopolymer-based hydrogels such as CBHs are suitable due to their better functionality, good solubility in organic solvents, enhanced surface area, abundancy, low-cost, better adsorption capacity, biodegradability, and ease of fabrication and recyclability. Additionally, the excellent hydrophilicity makes the CBHs a promising adsorbent for wastewater treatment [71,72,73]. However, the performance of an adsorbent for the removal of pollutants from wastewater is highly selective to the physical and chemical properties of the adsorbent materials [47]. Therefore, the first and foremost step for an effective adsorption process is to synthesize a suitable CBH adsorbent having high adsorbability to the dyes and heavy metal ions present in wastewater streams.

CBHs are typically derived from cellulose (i.e., native/pure cellulose and bacterial cellulose), its derivatives (ether derivatives: methylcellulose, ethylcellulose, hydroxyethyl methylcellulose, hydroxypropyl cellulose, carboxymethyl cellulose (CMC), etc.; ester derivatives: acetate trimellitate, acetate phthalate, hydroxypropyl methyl phthalate, hydroxypropyl methyl phthalate acetate succinate, etc.), and/or its composites (such as polyelectrolyte complex, interpenetrating polymer networks and blending with other polymers) [48,74]. One of the greatest challenges of HG synthesis from these base materials is dissolution in a common solvent; while some of the cellulose derivatives are water-soluble, native/pure cellulose is hardly soluble in most common organic and inorganic (e.g., water) solvents. Consequently, a suitable solvent is a prerequisite for CBH synthesis that includes alkali/urea (or thiourea), LiCl/dimethylacetamide, N-methyl morpholine-N-oxide, and ionic liquids. 

**Figure 1:**
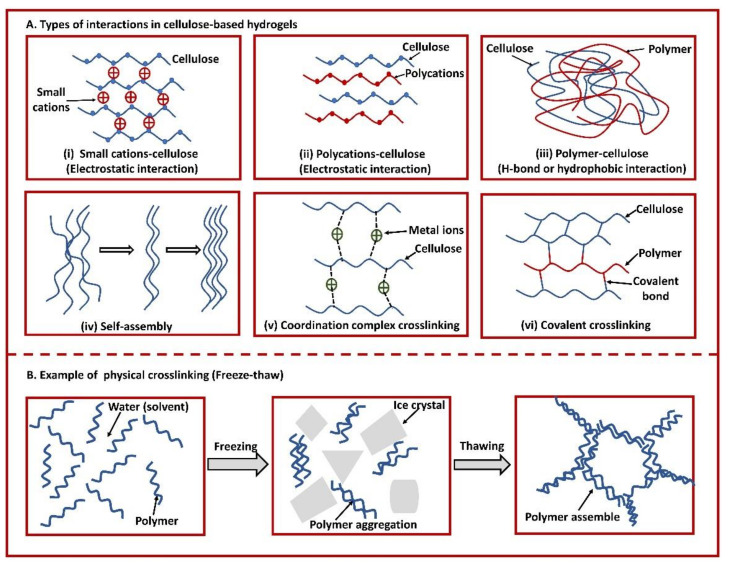
Illustration of (**A**) interactions in cellulose-based hydrogels in different systems—physical crosslinking (**i**–**v**) and chemical crosslinking (**vi**), and (**B**) an example of physical crosslinking (freeze–thaw). (**A**) (**i**) Electrostatic interaction between small cations and cellulose chain; (**ii**) electrostatic interaction between opposite charges of polycation molecule and cellulose chain; (**iii**) H-bond or hydrophobic interaction between polymer molecule and cellulose chain; (**iv**) self-assembly of cellulose molecules to fold into scaffolds by weak non-covalent bonding mechanisms—including hydrogen bonds, van der Waals forces, and hydrophobic interactions; (**v**) coordination complex crosslinking between multivalent metal ions and cellulose chain; and (**vi**) covalent crosslinking among functional moieties of cellulose chains and/or polymer chains, sometimes with the help of crosslinkers. (**B**) Fabrication of hydrogels through physical crosslinking by freeze–thaw method.

A stable 3D network structure of HG that is critical to hold a huge amount of water in their interstitials is achieved by crosslinking the constituting polymer chains [74,75]. Cellulose has hydrophilic functional groups, such as hydroxyl (−OH), that enable them to form both physical crosslinking (electrostatic interaction) and chemical crosslinking (covalent interaction using crosslinker). Depending on the materials used and modes of fabrication, different types of interactions are observed in CBHs, such as (i) small cations–cellulose (electrostatic interaction), (ii) polycations–cellulose (electrostatic interaction), (iii) polymer–cellulose (H-bond or hydrophobic interaction), (iv) self-assembly, (v) coordination complex crosslinking, and (vi) covalent crosslinking [76]. Figure 1A illustrates different types of interactions between cellulose molecules and other polymers or small molecules within a hydrogel matrix. All these interactions lead to the formation of the crosslinked structure of the hydrogels that prevents the complete destruction and dissolution of the CBHs during swelling. Cellulose-based hydrogels can be designed in a variety of physical shapes, including spherical, cylindrical, bead, blocks, microparticles, nanoparticles, and films by diverse methods of fabrication. 

**Figure 2:**
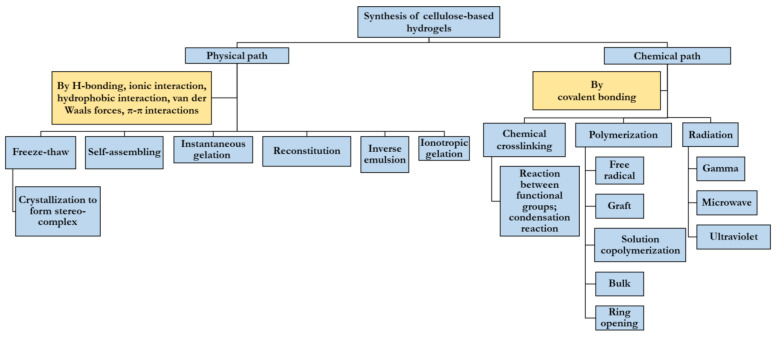
Physical and chemical paths of cellulose-based hydrogel (CBH) synthesis.

Figure 2 shows different pathways of synthesis of CBHs. Supplementary Materials Tables S1 and S2 include recent works on heavy metal and dye removal using CBHs along with their adsorption capacities.

### 2.1. Physical Path of Crosslinking

However, physical crosslinking process held by weak connections such as hydrogen bonds (H-bond), ionic interactions, hydrophobic interactions, π–π interactions, and van der Waals forces [72,77,78,79,80,81,82,83] is often more favorable for ecofriendly and non-toxic HG synthesis, as it does not involve use of chemical-based crosslinking agents [84] Although CBHs developed by the physical crosslinking process suffer from poor mechanical properties, they are widely used for adsorption purposes because of their high porosity having a higher chance to adsorb more pollutants, low sensitivity to pH, ease of regeneration (being reversible process), and no reduction in adsorption capacity due to potential reaction with a crosslinker as in the chemical process [60,85,86]. These HGs are prepared in the following methods. 

#### 2.1.1. Freeze–Thaw

One of the physical methods to prepare HGs is crystallization by the freeze−thaw technique [83]. In the freeze–thaw technique, crystallization occurs by freezing bulk solvents or low molecular solutes that increase the concentration of polymer by minimizing the chain space in the polymer and enable the chains to align and connect to each other to form a network structure (illustrated in Figure 1B) [75,87]. Freeze−thaw cycles allow a porous structure to be created in HGs because of the space left from the melting crystals at thawing stages [87,88]. The mechanical properties of the freeze−thawed HGs can be modulated by altering the concentration of polymers, the number of freeze−thaw cycles, the freezing and thawing time, and the freezing temperature [89].

HGs prepared by the freeze−thaw process show better elastic properties than that of the HGs prepared by chemical methods and hence drawing large-scale attention around the world [90]. Bio-compatible and non-toxic polysaccharide-based (such as cellulose, dextran, pullulan, and carboxymethyl curdlan, CMC, etc.) HGs are developed following this process [91]. For example, PVA (poly vinyl alcohol)/CMC HGs are prepared by freeze−thaw processes and applied to adsorb heavy metal ions including Ag^+^, Ni^2+^, Cu^2+^, and Zn^2+^ [87].

#### 2.1.2. Self-Assembling

The idea of using self-assembled CBHs preparation has grabbed the attention of researchers, as it does not need any crosslinkers to prepare. Self-assembling HGs are developed when the constituting monomers in the form of fibrils are assembled via non-covalent interactions (van der Waals forces, electrostatic interactions, hydrogen bonding, and π–π stacking interactions) followed by the entanglement of the fibrils into a robust network [92]. The mechanical properties of these HGs can be tuned by changing the concentration of the constituting monomers. Cationic guar gum (CGG) and TEMPO (2,2,6,6-tetramethylpiperidine-1-oxyl)-oxidized cellulose nanofibers (TOCN) can instantaneously form HGs as soon as they come in contact with each other. Formed HGs can adsorb metal ions as well as dyes from wastewater. They can also be used to treat oily wastewater by coating filter papers in a layer-by-layer deposition process [71]. 

#### 2.1.3. Instantaneous Gelation

The instantaneous gelation method is another way to develop HG instantly following the one-step method [93,94]. Magnetic chitosan-based HG beads (m-CS/PVA/CCNFs), consisting of carboxylated cellulose nanofibrils (CCNFs), amine-functionalized magnetite nanoparticles, and poly (vinyl alcohol) (PVA) blended chitosan, have been prepared by the instantaneous gelation method as adsorbents for Pb(II). The prepared HG beads can be persevered in distilled water for future use and recovered with the aid of a magnet if required [51]. 

#### 2.1.4. Reconstitution

Cellulose-based composite HGs are prepared by the reconstitution method that often uses ionic liquid as a solvent. Here, the composite HG is held by a strong intermolecular H-bond that eventually contributes to the tensile strength of the developed HG. For instance, biodegradable collagen/cellulose HG beads (CCHBs) have been prepared by reconstitution from a 1-butyl-3-methylimidazolium chloride ([C_4_mim] Cl) solution that can potentially be used to adsorb both dyes and heavy metals [95].

#### 2.1.5. Inverse Emulsion Technique

Water-in-oil emulsion refers to a phenomenon that occurs when water droplets are dispersed in oil (the continuous phase: paraffin oil) by using a suitable stabilizing agent (such as non-ionic surfactant Triton X-100), and then the system undergoes phase inversion in a coagulation bath to leach out the droplets and precipitates the porous film. This mechanism is widely known as the inverse emulsion technique. Partially hydrolyzed polyacrylamide grafted Arabic gum (AG-g-PAM/PAA)-based HG has been prepared by the inverse emulsion technique that can be used for dye and metal ion adsorption. This HG showed the adsorption of Methylene Blue (MB) with a capacity of 200 mg/g from an aqueous solution. The results revealed an ability of the novel porous HG to adsorb 99% of dye in only 10 min [96]. In addition to that, HGs based on sodium carboxymethyl cellulose and acrylic acid were prepared by inverse emulsion polymerization using potassium persulfate as an initiator and N,N′-methylenebisacrylamide as a crosslinker. The maximum swelling capacity for the HGs was 544.95 g/g in distilled water and 44.0 g/g in 0.9% *w*/*v* NaCl solution [97].

#### 2.1.6. Ionotropic Gelation

The ionotropic gelation (IG) technique allows the production of nanoparticles and microparticles by electrostatic interactions between two ionic species under certain conditions where at least one of the species should be a polymer [98]. CBH beads are successfully synthesized by the ionotropic gelation of sodium alginate (SA) and hydroxypropyl cellulose (HPC) solutions with varying ratios (0:50, 75:25, and 100:0) followed by extrusion through a syringe to form HG beads. The adsorption property of the produced bead is largely influenced by the concentrations of SA and HPC. The beads showed 47.72 mg/g adsorption capacity and a 95.45% adsorption percentage of Pb^2+^ [99]. Cellulose nanocrystals and alginate-based HG beads are also synthesized by the ionotropic gelation that can be used for the adsorption of organic dyes [100].

### 2.2. Chemical Path of Crosslinking

Chemical crosslinking is preferred when high mechanical strength is required through introducing a chemical crosslinking agent forming strong molecular bonds such as via covalent and electrostatic interactions. However, the degree of crystallinity negatively impacts the adsorption capacity and swelling ratio due to the reduced pore size and rigidity of polymer chains [61]. In addition, crosslinking agents and the higher crosslinking density engage some binding sites of the adsorbent; therefore, there should be a balance between maintaining the required mechanical strength and maximizing the adsorption capacity [60,61]. The chemical path of crosslinking can be achieved in the following ways.

#### 2.2.1. Crosslinking by Chemical Reaction

In chemical crosslinking, the bond is formed between the crosslinking agent and polymer or among the functional groups of polymer molecules. A polymer having hydroxyl (such as cellulose and its derivatives) and amine (such as chitosan, proteins) groups are connected with crosslinker having an aldehyde group (aldol formation) via covalent bond [75]. HG formation for the polymers with –OH groups requires specific conditions such as lower pH, high temperature, and methanol as a quencher, whereas protein-based HGs can be formed in normal conditions [61,101,102,103]. A typical crosslinker for CBHs includes glutaraldehyde and epichlorohydrin, formaldehyde, acetaldehyde due to their availability, and cost-effectiveness [61,75,104]. For instance, environment-friendly CBH beads have been produced from CMC by using an epichlorohydrin (ECH) crosslinker in an inverse suspension (fluid wax) crosslinking mechanism, where ether linkage is developed between ECH and CMC contributing to high porosity in the structure of HG. More specifically, the porous structure of the HG is attributed to the presence of numerous carboxylate anions (–COO^−^) in a network of HG beads, which not only helps expand the HG network but also increases the size and amount of pore enabling more metal ion to anchor [105]. Ge et al. also prepared a high-performance composite hydrogel (cellulose/poly-ethylene imine (PEI)) by grafting hyperbranched PEI onto cellulose chains using an ECH crosslinker [106]. The hydrogels showed excellent Cu^2+^ ion removal capacity (ca. 285.7 mg/g) and good stability over a wide range of pHs and temperatures due to the chemical crosslinking facilitated by ECH. Similarly, in the presence of a crosslinker, condensation and additional reactions occur between polymers having amine and ester moieties, and a crosslinker, causing Schiff base formation [107].

#### 2.2.2. Crosslinking by Polymerization 

A large number of CBHs are synthesized by the chain growth polymerization process, which is accomplished in three steps including initiation, propagation, and termination [49]. Among different types of polymerization processes, free radical is characterized by a faster synthesis process, ease of implementation under various reaction conditions, wide-ranging temperatures, and low costs, and it is widely followed for HG preparation [75,108]. Here, for the initiation step, a free radical is generated from the initiator such as potassium persulphate (KPS), tetramethylene-diamine (TEMED), ammonium persulfate (APS), etc., in the presence of some conditions in term of light, pressure, temperature, and radiations. In propagation, polymer chain growth occurs, and the crosslinker reacts with the growing polymer chain, randomly leading to forming a 3D network structure, followed by the termination of the polymerization via combination or disproportion [49,60,61]. Cellulose-graft-acrylic acid (C-g-AA) HGs are prepared via free-radical polymerization in 85% phosphoric acid solution in the presence of 50 mg ammonium persulfate (APS) as an initiator and 10 mg N,N′-methylene bisacrylamide (MBA) as a crosslinker. Acrylic acid acts as a grafting monomer and can bind heavy metal ions and dyes through its carboxyl groups. The mechanism behind the HG preparation is the extraction of hydrogen atoms from the cellulose molecules to produce cellulose macro-free radicals on the cellulose chains using peroxodisulphate (S_2_O_8_^2–^) [52]. In another study, a similar type of hydrogel was prepared from cellulose and CMC separately or in a mixture of both of them by polymerization with partially neutralized AA [109]. The polymerization was initiated by KPS, and vinylsulfone (VS), glutaraldehyde, MBA, and ECH were used as crosslinkers. The hydrogel was successfully used to remove and recover heavy metals such as Cu(II) from wastewater. The addition of a modifier such as tannic acid (TA) to cellulose-based hydrogel can be helpful to attain a homogeneous pore structure of the hydrogel that improves the adsorption performance. Ning et al. [110] synthesized an HEC-co-p(AA-AM)/TA (HEC: hydroxyethyl cellulose, and AM: acrylamide) hydrogel by the grafting of AA and AM onto HEC, followed by modification with TA. The hydrogel showed excellent MB adsorption performance (ca. 3438.27 mg/g) with high reusability. In a recent study, a novel fluorescent lignin-based hydrogel with cellulose nanofibers and carbon dots (CDs) was synthesized by free radiacal polymerization [111]. The hydrogel demonstrated 3D porous structures that provided many active sites and ion transport channels, thereby improving the adsorption performance for hexavalent chromium(Cr(VI)) (maximum adsorption capacity 599.9 mg/g).

#### 2.2.3. Crosslinking by Radiation

It is also a polymerization process, where no chemical-based crosslinker or catalyst is used. Instead of a chemical crosslinker as in the usual polymerization process mentioned above, it induces different types of radiations (such as gamma, electron beams, microwave, and ultraviolet radiations) to crosslink the polymer chains. Therefore, it is an environmentally benign synthesis process involving zero waste generation [60,61]. CMC-Na is a widely used derivative of cellulose, which is synthesized by gamma irradiation [53].

## 3. Adsorption Mechanism

**Figure 3:**
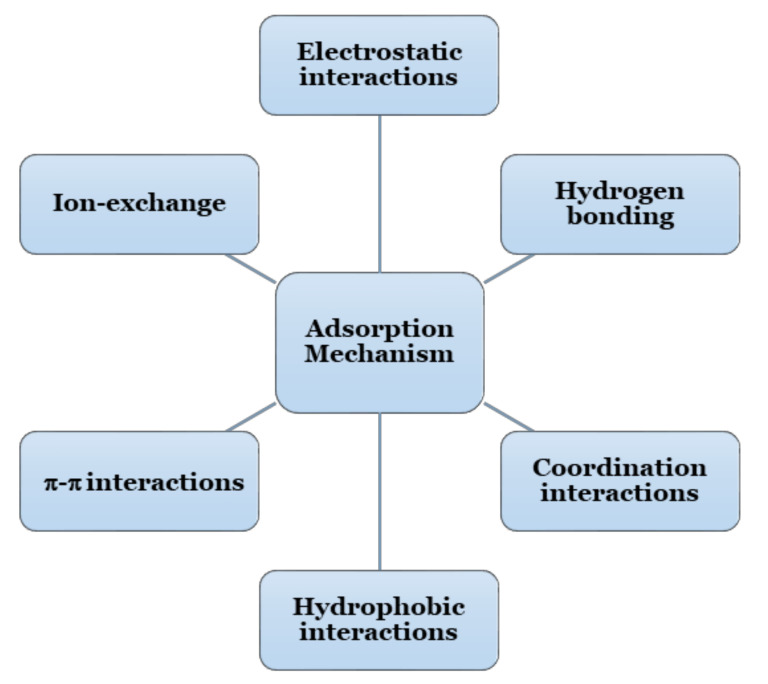
Adsorption mechanism of dyes and heavy metals by CBHs.

**Figure 4:**
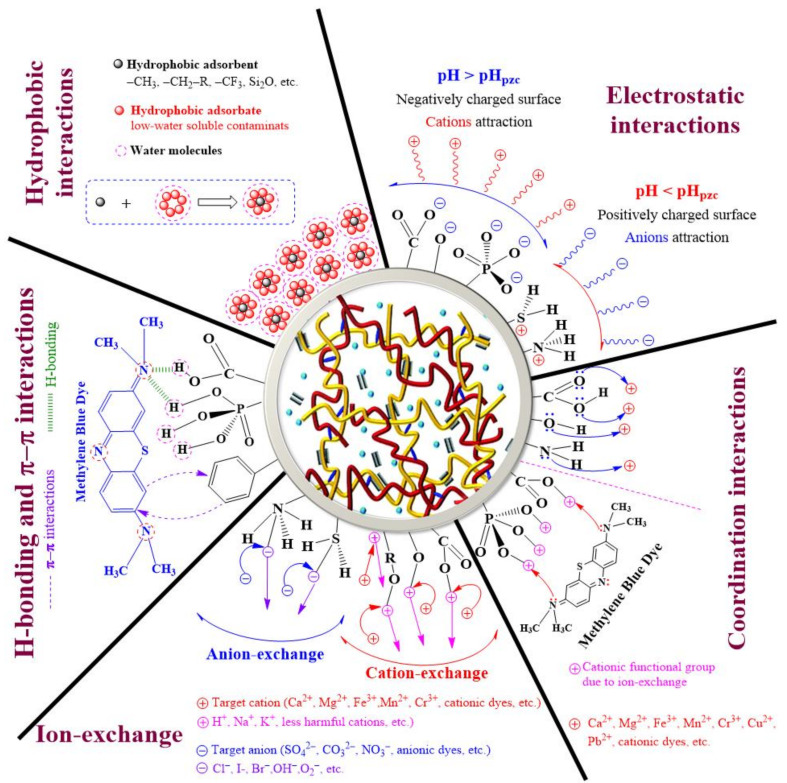
Adsorbent–adsorbate interaction mechanisms for the decontamination of wastewater by CBHs.

A comprehensive understanding of the adsorption process along with the removal mechanism of several pollutants on respective CBHs is very indispensable for further modification of CBHs to improve their adsorption performance. The adsorption by CBHs typically occurs through different types of interactions, which are extensively dependent on the functional groups present in the HG, adsorbent properties, the chemical composition of pollutants, and experimental parameters (i.e., initial pollutant concentration, solution pH, temperature, the coexistence of metal ions, etc.) [60]. The most common adsorption mechanism for the removal of dyes and heavy metals by CBHs is electrostatic interactions; however, combinations of other interactions along with electrostatic interaction are also reported in many adsorption processes [105,112,113,114]. The different adsorption mechanisms of dyes and heavy metals by CBHs are shown in Figure 3 and Figure 4.

### 3.1. Electrostatic Interactions

Electrostatic interaction comprises the interaction between charged modules; attractive and repulsive interaction occurs when molecules are oppositely-charged (cation–anion interactions) and similarly-charged (cation–cation or anion–anion interactions), respectively [115]. To remove ionic contaminants electrostatically by CBHs, the adsorbent surface must oppositely be charged to the respective ions that need to be adsorbed. Based on the pollutant nature and chemical properties, the CBHs are synthesized with specific functional groups, which are capable of producing the opposite charge corresponding to target ions [60]. Additionally, the formation of charged species on the adsorbent surface intensely depends on the pH of the solution [116]. pH_PZC_ represents the pH of the solution when no charge species exist on the adsorbent surface [117,118,119]. At pH > pH_PZC_, functional groups, such as –COOH, –OH, and –H_3_PO_4_ are deprotonated due to an excessive concentration of OH^−^ in the aqueous solution that creates anions (such as –COO^−^,–O^−^, –PO_4_^3–^, etc.) on the adsorbent surface, resulting in attractive interactions between cationic contaminants and the anionic adsorbent surface (Figure 4). In contrast, the adsorbent surface is positively charged pH < pH_PZC_ due to the protonation of functional groups (i.e., –NH_2_, –SH, etc.) on the adsorbent surface as a consequence of an increase in H^+^ concentration in the solution. Here, a recent study also found the highest electrostatic interactions between a high-capacity HG with –NH_2_ groups and an anionic dye (Acid Black 1) at low pH conditions, which was decreased with rising pH and reached the least interactions for very high pH condition [112]. In another study, CMC adsorbed the utmost amount of Pb^2+^, Cu^2+^, and Ni^2+^ from aqueous solution through electrostatic interactions at higher pH [105]. Similar electrostatic nature was perceived for the removal of MB (a cationic dye) by chemi-mechanical pretreated cellulose-based superabsorbent hydrogel [97]. Hu et al. [120] also observed electrostatic adsorption with complexation between –OH and –COOH functional groups of sodium alginate–CMC–cellulose gel beads for the removal of Pb^2+^.

### 3.2. Ion Exchange

Ion exchange refers to an exchange of ions between a liquid (wastewater) and an insoluble solid (adsorbent). Unwanted dissolved ions (cations or anions) in an aqueous solution are removed and replaced with ions of the same charge on the adsorbent surface. In a perfect ion-exchange process, the number of ions released from the adsorbent surface is equivalent to the number of ions adsorbed by the adsorbent molecules [121]. Ion exchange is a very convenient and efficient tool especially for the removal of hazardous pollutants, such as dyes and heavy metals from wastewater. This process decreases the degree of hazardous load by transforming pollutants into a shape in which they can be recycled, leaving behind less harmful elements in their place or enable ultimate discharge by decreasing the hydraulic flow of the stream carrying toxic elements. In addition, the ion-exchange process has the capability to discrete as well as distillate contaminants [122]. Similar to electrostatic interactions, ion-exchange mechanism also shows a strong dependency on the pH of the solution. At pH < pH_PZC_, the functional groups of adsorbent are positively charged because of an increase in H^+^ concentration, resulting in cations exchange. On the other hand, functional groups are negatively charged when pH > pH_PZC_, which causes anions exchange (Figure 4) [123]. Zhou et al. [124] observed ion exchange and chelation between positively charged ions (Cd^2+^, Ni^2+^, and Pb^2+^) and ionized/non-ionized carboxylic groups within the HG during the removal of these metal ions from aqueous solutions using cellulose–graft–acrylic acid hydrogel at pH 2.5–6.0, since –COOH groups in the HG surface are protonated at lower pH, which replaced metal ions with H^+^ from –COOH groups. In another investigation, Ca^2+^ ions replaced cations from cellulose–graft–polyacrylamide/hydroxyapatite composite HG and attached to the hydroxyapatite surface through an ion-exchange mechanism [125]. Xiong et al. [126] developed a self-cleaning hybrid (cellulose–titanate) hydrogel microsphere by a simple sol–gel process that exhibits an excellent ability to remove heavy metal from oily wastewater. The strong physical and chemical interaction between titanate nanotubes (TNTs) and cellulose fibers helped inherit and integrate the intrinsic properties of both titanate and cellulose hydrogels. At first, the heavy metal ions (Cu(II)) were adsorbed on the inside of the hydrogel under the electrostatic interaction. Then, through ion exchange, Cu(II) ions were deeply trapped in the layer structure of TNTs. Thus, under the synergistic effects of physical and chemical adsorption, the hydrogel revealed excellent adsorption properties for heavy metal ions. Similar ion-exchange mechanisms have been found for the removal of dyes from aqueous solutions [127,128,129].

### 3.3. Hydrogen Bonding

H-bonding is a distinct form of dipole–dipole interaction that results from the electrostatic attractive force between a positively charged H-atom and a more electronegative atom (i.e., N, O, F, etc.) or group, which are covalently bonded [130]. During the treatment of dye-containing wastewater, functional groups having oxygen (i.e., –COOH, –OH) in the adsorbent molecules participate in H-bonding with pollutants (dyes) [131,132]. The adsorption of MB on a CBH (synthesized by modification of cellulose and acrylic acid) showed such interaction between electronegative N-atom in MB structure and H atom in –COOH and –OH groups of HG (Figure 4) [133]. Recently, Sekine et al. [70] developed eco-friendly CMC nanofiber HGs, which were used to remove numerous chemical dyes through hydrogen bonding, electrostatic interactions, and hydrophobic interactions between the functional groups of dye and adsorbent molecules. Lie et al. [46] extensively explained the H-bonding interactions between the sulfur (S) atoms in both anionic (Acid Blue 93) and cationic (Methylene Blue) dyes and the H atoms in a CBH material along with the graphical presentation.

### 3.4. Hydrophobic Interactions

Hydrophobic interaction defines the interaction between hydrophobes and water molecules. Hydrophobes are non-polar compounds, which are composed of long chains of carbon that cannot interact with water molecules due to weak van der Waals attractive forces [134]. In addition, low water-soluble elements show a high tendency to be attracted to hydrophobes. Therefore, during wastewater treatment, hydrophobic interactions are formed to remove non-polar pollutants (i.e., pigments, disperse dyes, organic compounds, etc.) from aqueous solution (Figure 4) [135]. Li et al. [136] demonstrated both electrostatic and hydrophobic interactions between MB dye and functional groups (–SH and –OH) present in thiol-modified CMC/L-cysteine HG. Hydrophobic interactions in CBHs offer extensive opportunities in HG engineering because of their roles in enriching mechanical properties [137,138]. Furthermore, the domain of hydrophobes provides a physical crosslinking point with optimal mechanical stiffness during the initiation of chemical reactions. The reaction continues with the formation of interactions between crosslinking points and other polymeric chains until macromolecular three-dimensional polymer networks are formed [139]. Lazzari et al. [140] recently showed that hydrophobic interaction is one of the main driving forces to adsorb insoluble organic pollutants into cellulose cryogels. However, CBH is often modified with both hydrophilic and hydrophobic functional groups to remove pollutants from aqueous solutions. The hydrophilic part of functional groups attracts soluble ionic pollutants through either electrostatic, H-bonding, and/or ion-exchange interactions, while the hydrophobic part contributes to the adsorption of water-insoluble contaminants [141,142]. 

### 3.5. Coordination Interactions

The coordination interaction refers to a covalent bond wherein both electrons are shared by a single atom. In the removal of cations (heavy metal ions and/or cationic dyes) from wastewater through a coordination mechanism, cations attract atoms in functional groups that have lone pair electrons (i.e., O and N) in outer orbitals, resulting in the adsorption of cations on the adsorbate surface. Coordination interactions can also happen along with other interactions including ion-exchange, electrostatic interactions (Table 3). For example, at low pH, H^+^ ions from the adsorbent surface have been replaced with metal ions present in the solution, and these metal ions have a high affinity to negative electrons. Typically, different metal ions as well as N and O atoms in dye molecules are adsorbed on ion-exchanged functional groups (i.e., –OH) via coordination bonds (Figure 4). One such experiment was recently conducted by Jayabrata Maity and Samit Kumar Ray (2017), where they observed the combined effects of coordination and electrostatic interactions during the removal of Cu^2+^ using sugar cane bagasse cellulose and gelatin-based composite hydrogels. Cu^2+^ ions formed coordination bonds with N or O atoms, which were sourced from –NH_2_ and –OH functional groups, respectively [143]. Tang et al. [54] also observed coordination interactions between metal ions and the O atoms (from –OH group) during the removal of Hg^2+^, Pb^2+^, and Cu^2+^ using chitin/cellulose composite (3:1) adsorbent. The adsorption mechanism of chitosan/cellulose composite adsorbent for the removal of Congo Red (CR) dye revealed electron sharing (coordination interaction) and transfer (electrostatic adsorption) between the adsorbent and adsorbate molecules [55].

### 3.6. π–π Interactions

π–π interaction is a non-covalent interaction between adsorbent and adsorbate molecules in an aqueous solution. Numerous chemical properties, such as chemical bonding, boiling points, molecular and biomolecular crystallography, the structure of π-adjacent molecules, etc. are widely affected by π–π interactions [157]. In typical π–π interaction, at least one of the molecules contains a π electron-rich or a deficient group in the structure of benzene or other aromatic rings that causes interactions in an aqueous medium. The π–π interaction significantly depends on the functional groups present on both the adsorbate and adsorbent surface and medium of the solution (pH) [158]. Based on these functional groups coupled with the pH of the solution, the adsorbate and adsorbent molecules act as an electron-donor or electron-acceptor, resulting in forming various π–π interactions (electron-donor-acceptor, electron-acceptor-acceptor, and electron-donor-donor) [159]. These types of interactions are usually found during the adsorption of organic pollutants and dyes onto graphene-, graphene oxide-, or carbon-based HGs. Sharma et al. [153] attained an adsorption capacity of 96.43 mg/g for Methyl Violet (dye) using a CMC-structured nano adsorbent. π–π stacking with electrostatic interactions were reported as the potential causes for such high adsorption capabilities where contaminants donated π-electrons to the adsorbent molecules. Chen et al. [160] also reported that π–π stacking is the primary driving force in the removal of heavy metal ions (Cu^2+^, Zn^2+^, Fe^3+^, and Pb^2+^) from wastewater using GO/cellulose HG. The –COOH and –OH functional groups on the adsorbent surface (introduced from GO) made π–π interactions with adjacent metal ions. The role of GO in the HG was to enhance mechanical strength as well as the adsorption capability of porous GO/cellulose HGs. Another type of GO/cellulose HG with high mechanical and thermal stabilities was prepared to remove contaminants from waste solution [161]. In another study, Yan et al. [162] prepared a self-healing HG with great mechanical strength based on cellulose-derived co-polydopamine@Pd nanoparticles for the reduction of contaminant dye in wastewater. They attained >95% removal of both anionic and cationic dyes from wastewater through π–π interactions, hydrogen bonding, and coordination interactions without a significant decrease in the performance or integrity of the HG structure, even though the water molecules continuously weaken to van der Waals interaction to decrease the mechanical properties and stability of HG [163,164,165]. For carboxymethyl cellulose sodium (CMCNa)/graphene oxide (GO) hydrogel microparticles, Liu et al. [166] suggested that the adsorption mechanism for dyes were due to both electrostatic and π–π interactions, while those for heavy metals were the synergistic effect of electrostatic interactions, surface complexation, and ion exchange.

## 4. Factors Affecting the Adsorption Capacity of CBHs

### 4.1. Crosslink Density

**Figure 5:**
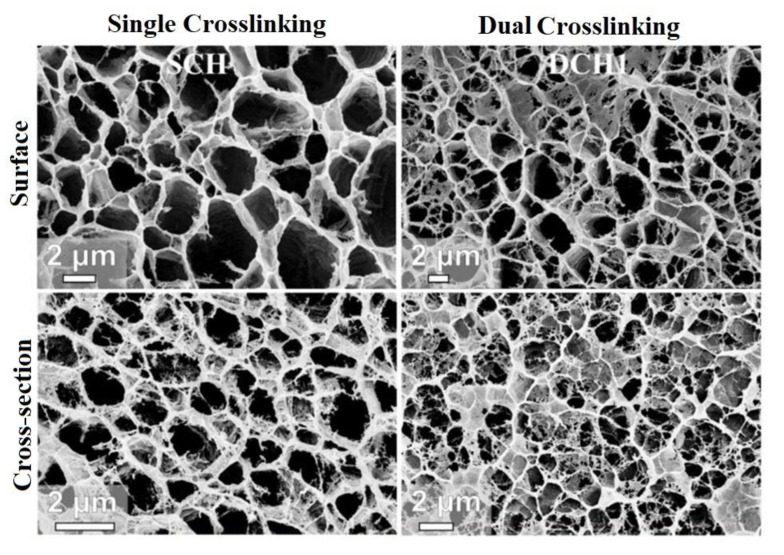
Morphology of single crosslinked hydrogel (HG) (SCH) and dual crosslinked HG (DCH) specimens: Surface and cross-sectional SEM images of SCH and DCH [171].

Crosslink density refers to the density of chains or segments that attach two or more parts of the polymer network, instead of the density of crosslink junctures. The adsorption capacity of CBHs is highly affected by the crosslink density of polymeric segments [167,168,169]. The highest adsorption capacities for CBHs are typically obtained for the lowest crosslink density and vice versa [168]. However, there is a minimum value of crosslink density, which is necessary to avoid mechanical failure or outright dissolution of the adsorbent materials [170]. At the point of the lowest crosslinking density, the molecular weight of the HGs is preferred to be high to ensure that most of the polymeric chains are bound by a minimum of one covalent bond to the rest of the material. Studies showed that higher molecular weight CMC HGs have a higher internal volume in the polymeric chain that causes an increase in the adsorption capacity of the HGs [169]. The surface and cross-section porous structure of a CBH is shown as a function of crosslinking density in critical content. The crosslink density of dual crosslinked hydrogel (DCH) was higher than single crosslinked hydrogel (SCH) because of a decrease in water content during crosslinking. Due to the increase of crosslink density in the polymeric network, the adjacent chains of HG materials came closer, resulting in a significant decrease of the surface and cross-section porous structure of DCH (Figure 5) [171].

### 4.2. Initial Concentration of Pollutant (ICP)

The amount of pollutants adsorbed on the adsorbent surface is highly dependent on the initial concentration of pollutant (ICP). In a fixed solution volume and adsorbent mass, the number of adsorbate molecules is proliferated when ICP in wastewater is increased [172]. Consequently, more adsorbate molecules bind to the active sites of the adsorbent, thus accelerating the diffusion of dyes or heavy metals onto the adsorbent sites due to the increase in driving force of concentration gradient, resulting in higher adsorption capacities [9]. However, a decline of adsorption efficiency due to higher pollutant concentration was reported in some recent works [173,174]. In the typical adsorption process, adsorption capacity is sharply increased until the plateau state. Afterward, a further increase in ICP does not improve the adsorption process, resulting in a decrease in adsorption capacity [175]. The following explanation was made in most of the current works: at low pollutant concentration, the ratio of an initial number of moles of pollutant ions to the accessible sites of CBH is large, which causes higher adsorption capacity. On the other hand, at higher pollutant concentrations, the number of available adsorbent sites becomes fewer, resulting in a decrease in pollutant removal efficiency [160,174]. Wang et al. [155] studied the influences of the initial concentration of MB dye on adsorption capacity utilizing MB concentration between 1800 and 2700 mg/L. The adsorption capacity was linearly increased with initial dye concentration until a plateau was achieved at 2500 mg/L. At a concentration above 2500 mg/L, the adsorption capacity started to decrease with increasing concentration. Recent studies on other CBHs for the removal of dyes and heavy metals also reported a similar trend of adsorption capacity with respect to ICP. The adsorption capacities of different CBHs as a function of initial concentration are summarized in Table 4.

### 4.3. pH at the Point of Zero Charge

The pH at the point of zero charge (pH_PZC_) is a critical parameter for the adsorption process that can change the chelating ability of adsorbents by affecting their swelling ability and interactions between adsorbents and ions [156]. When the pH < pH_PZC,_ the adsorbent surface is positively charged because of an increase of H^+^ concentration in the aqueous solution (protonation). Hence, strong electrostatic interactions occur between the positively charged adsorbent surface and anions. Conversely, the aqueous solution is deprotonated at pH > pH_PZC_, creating a negatively charged surface that interacts with cations [117,118,119]. The protonation and deprotonation mainly occur at different functional groups, such as carboxylic [105] or amine [174,178], and the precipitation of ions in HGs. Typically, the adsorption capacity of CBHs for heavy metal ions is higher at the basic pH of the solution; in fact, there is a range of pH values for each metal ion wherein the maximum adsorption occurs. However, at pH > 7.0, metal ions interact with excess OH^−^ in aqueous solution and precipitate as metal hydroxides form, thus impeding the adsorption process and reduce the adsorption capacity of HGs. The typical pH used for optimum metal adsorption on CBH materials is about 5.0–6.0 [160,178,179]. Recently, Amr El-Hag Ali [156] investigated the adsorptive nature of CMC as a function of solution pH for the removal of heavy metals such as Co^2+^, Cu^2+^, Fe^3+^, and Mn^2+^ from wastewater. Results showed that the adsorption capacity of CMC HGs increases at higher pH values. Excessive H^+^ at extremely low pH values compete with metal ions to cohere active sites of the CMC, causing a lower uptake of metal ions. The same adsorption protocol is applicable to eliminate dyes from wastewater using CBHs. For instance, the maximum adsorption capacity of a novel CBH for the removal of Congo Red (CR), an anionic dye, was obtained at pH ≈4.0, and the adsorption capacity was declined with increasing pH values. At higher pH, the excess OH^−^ covers active sites of adsorbent molecules that limit the adsorption of CR dye molecules [82]. Conversely, cationic dyes are adsorbed on the adsorbent surface at higher pH values such as metal ions [116]. Table 5 includes some examples of pH values required to maximize the adsorption capacity of various CBHs. 

### 4.4. Temperature

**Figure 6:**
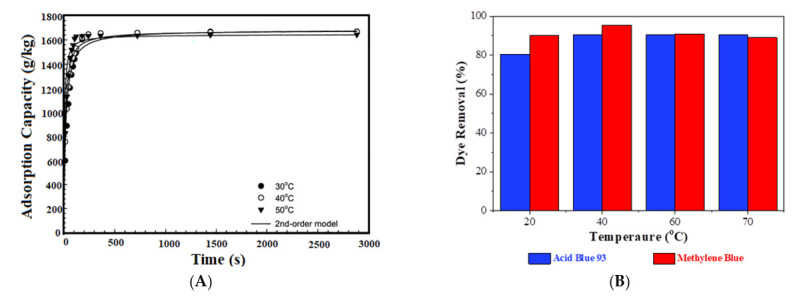
Effect of temperature on adsorption capacity: (**A**) adsorption kinetics of Reactive Red 189 dye on crosslinked chitosan; pH = 3.0, initial dye concentration = 3768 g/m^3^, adsorbent size = 2.3–2.5 nm, and crosslinking ratio = 0.2 [184], (**B**) percent adsorption of Acid Blue and MB dyes on CBHs [46].

The adsorption capacity of CBHs greatly depends on temperature since the kinetic of adsorbate molecules in the aqueous solution is significantly changed when the temperature is raised. In the endothermic adsorption process, the adsorption capacity of CBHs is increased with the increase of temperature, which is the opposite of exothermic adsorption [100,182]. Typically, the adsorption capacity of CBHs for the removal of dyes from an aqueous solution is proportionally increased with temperature [46,183,184]. According to Lin et al. [46], the mobility of dye molecules is notably increased at higher temperatures, providing a higher potential to enhance the interactions between dye molecules and the adsorbent surface. As a result, the dye desorption from the adsorbent surface is minimized, leading to a higher adsorption capacity. Additionally, a swelling effect within the CBH structure may initiate due to the increase of temperature, causing the further penetration of dyes onto the adsorbent surface [182]. However, the adsorption capacity becomes independent of temperature when equilibrium is achieved (Figure 6B; blue bar). Similar to non-CBH adsorbent (Figure 6A), the adsorption capacity of CBH adsorbent is slightly fallen with an additional increase of temperature after equilibrium (Figure 6B; red bar) [184]. When adsorption takes place at a temperature higher than equilibrium temperature, the desorption characteristics of the CBH molecules become dominant, owing to the excessive molecular motion that causes such a slight decrease of the adsorption capacity [46]. Furthermore, similar to adsorptive removal of dyes from wastewater, CBHs show higher adsorption capacity for the removal of metal ions through endothermic adsorption [185], while the adsorption capacity is significantly lower for exothermic adsorption [186].

### 4.5. Ionic Strength 

The ionic strength of an aqueous solution refers to the concentration of ions present in that solution. The ions in the solution are usually formed by dissociation of salts when dissolved in aqueous medium. In other words, the more salts in a solution, the higher the ionic strength of that solution [187]. Typically, industrial wastewater contains a wide variety of salts, such as NaCl, KCl, NH_4_Cl, CaCl_2_, MgSO_4_, AlCl_3_, etc. along with other organic and inorganic pollutants, and these salts show strong influences on the adsorption capacity of CBHs during wastewater treatment [188]. The relationship between ionic strength and adsorption capacity is mostly studied for dye removal from aqueous solutions. Liu et al. [46] demonstrated the effects of ionic strength on the dye removal efficiency of acrylic acid and acrylamide grafted CBH, where they used a different amount of NaCl salt to change the ionic strength of the solution. According to their study, the dye removal efficiency was decreased with increasing NaCl concentration, due to the competitive effect between the salt ions (Na^+^ and Cl^−^) and the existing dyes with functional groups (–COO^−^, –NH_3_^+^, and OH^−^) on the CBH surface. With the growth of NaCl concentration, the shielding effect of Na^+^ and Cl^−^ ions for the ionized dye molecules was improved, which causes the reduction of adsorption efficiency of adsorbents [189,190]. In addition to NaCl, the absorbency of CBH was also decreased with a higher concentration of other salts, including KCl, NH_4_Cl, CaCl_2_, and AlCl_3_. When compared among these salts, the adsorption capacity of CBH in the existence of monovalent cations reduces in the following order: NH^4+^ > K^+^ > Na^+^. However, when compared based on ion valance, the highest declination of the swelling capacity of CBH was observed in the presence of trivalent cations with the following descending order: trivalent > divalent > monovalent cations [191].

### 4.6. Coexistence of Ions

The coexistence of various ions in the aqueous solution has mixed impacts on the adsorption efficiency of HG for each ion species. When multiple ions present in the solution, some of the ions either decrease the adsorption of others due to competition or collectively increase the adsorption through cosorption [192,193]. Antic et al. [193] investigated the sorption of Pb^2+^ in the presence of other ions, including Ni^2+^, Cd^2+^, Cu^2+^, Zn^2+^, and Co^2+^. In the binary system, the adsorption of Pb^2+^ was decreased by 5.27% due to competition, and it further decreased to 11.1% when the tertiary system was used. Furthermore, the existence of interference ions such as K^+^, Na^+^, Mg^2+^, and Ca^2+^ in the solution causes competition with heavy metal ions, such as Cd^2+^, Cu^2+^, and Pb^2+^ around the same surface of the adsorbent molecules, impeding the adsorption of heavy metals [194]. Sharing the same binding sites of HGs by existing ions in the solution is another potential cause for such low adsorption of heavy metals [192,195]. 

Moreover, the functional groups (i.e., –COOH, –NH_2_, etc.) entrapped in HG polymeric chains have shown selective adsorption for certain ions based on ion properties, including ionic radius, electronegativity, and ionization potential. For instance, –COOH in a nanocomposite HG has higher adsorption selectivity to Pb^2+^ compared to Ni^2+^, Cd^2+^, Cu^2+^, and Zn^2+^ due to the difference in the above ionic properties [196]. The metal ions show more propensity to HGs compared to other ions when the radius of metal ions is relatively higher. So, the bigger the ionic radius, the superior the binding capacity [193,195]. In addition to ionic radius, ion hydration radius also plays a vital role in metal ions removal efficiency and adsorption capacity. The literature showed that the change in adsorption capacity is very minimal for the ions with smaller hydration radius, even though the solution consisted of different interfering ions such as Na^+^, Ca^2+^, and Ba^2+^ [197].

In addition to that, the alternation of functional groups in the CBH structure has great influences on the adsorption selectivity of the adsorbent for specific metal ions. Amr El-Hag Ali investigated the simultaneous adsorption of Mn^2+^, Co^2+^, Cu^2+^, and Fe^3+^ on CMC/2-acrylamido-2-methyl propane sulfonic acid (AMPS) HG derived by γ-radiations-induced copolymerization and crosslinking. He functionalized the adsorbent structure by altering the ceoncentratio of the AMPS. The uptake of metal ions for various AMPS concentration is summarized in Table 6 that revealed that the adsorption capacity of the CMC/AMPS adsorbent significantly increased with increasing concentration of the AMPS. One of the reasons for such behavior is solution pH. Typically, the pH of the solution changes when the functional groups in CBH structure are altered [198]. The upsurge of AMPS concentration in the HG directs to the increment in the dissociated groups and subsequently risese the electrostatic repulsion, resulting in the expansion of the network structure.

## 5. Conclusions and Future Outlook

Dyes and heavy metals released by various industries are among the most common pollutants of wastewaters, which have a detrimental effect on the environment including aquatic lives, human health, and the ecosystem. Therefore, the remediation of pollutants from the wastewater is imperative for a safer environment. The adsorption process involving different types of adsorbents is considered as an effective and efficient wastewater treatment method. However, most of the adsorbents used for the treatment purpose are synthetic and non-biodegradable, and management of the adsorbent after the treatment is another environmental concern that triggers the researchers to find and use a naturally derived and renewable source of materials as adsorbent. Cellulose-based hydrogels (CBHs) are ideal candidates meeting the requirement with some added benefits such as high removal efficiency, cost-effectiveness, and easy process. This review covers important aspects of wastewater treatment using CBHs such as the synthesis of CBHs, adsorption mechanism, and parameters to optimize adsorption capacities, which have barely been covered in the literature. In addition, based on the limitations in the literature covered, the following scopes are recommended to address for future research consideration.

- Most of the literature covered treatments of lab-based wastewater containing a single pollutant, instead of real industrial wastewater. Some reports claimed that the presence of multiple ions in the wastewater may influence the adsorption of any specific ions [192,193]. Some wastewaters contain additives or other auxiliaries (such as salts, surfactants, etc. in textile wastewater) besides the target pollutant, which might have some potential impacts on the adsorption efficacy, and hence need to be explored. Therefore, extensive future work is needed to investigate the adsorption performance of CBHs in the real industrial wastewater system. The primary attempt could be the pilot-scale treatment of the industrial wastewater or at least simulated wastewater containing multiple pollutants that mimic real industrial wastewater.
- The greatest limitation perceived by the authors during the preparation of the paper is the lack of clarity and inadequate information on the adsorption mechanism. Moreover, the adsorption behavior of CBHs for non-ionic pollutants such as non-ionic dyes, water-insoluble dyes (such as pigments, disperse dyes, vat dyes, sulfur dyes, etc.) have been overlooked in the literature. Consequently, more experimental and theoretical research is a pressing need to comprehend adsorption mechanisms that might potentially help unlock and identify the most effective mechanism.
- Many cellulose-based hydrogels lose their adsorption capacity after regeneration. Some reports revealed that the hydrogels retained the adsorption capacity only when regeneration is conducted with caustic soda. Moreover, modifications (both physical and chemical) and pretreatments of cellulose can enhance the adsorption capacity of the CBHs to some extent. Several chemical and physical networking approaches, such as modification with graphene oxide (GO), nanoparticles (NPs), carbon nanotubes (CNTs), and carbon quantum dots (CQDs), and blending with other suitable synthetic or natural polymers can be tested to enhance the gel characteristics as well as regeneration performances.
- The stable structure and effective swelling of CBHs are crucial for wastewater treatment especially at the condition of elevated temperature of the industrial waste stream. Some CBHs tend to weaken and lose their mechanical strength upon repeated swelling. A chemically crosslinked network often improves the stability and adsorption performance of the hydrogels. Moreover, the incorporation of magnetic particles, nanoparticles, and different chemical catalysts should be investigated to enhance the adsorption capacity and swelling properties of the CBHs. It is important to explore the scope of improving the mechanical durability of CBHs with increases in self-healing ability after a swollen state.
- The overall physiochemical composition and morphology of the CBHs mostly dictate their performance in the area of industrial wastewater treatment. A designed formulation and optimized synthesis conditions are critical parameters in designing a specific CBH overcoming the challenges and shortcomings such as low turnover number, lesser resistivity, and mechanical strength. In addition, issues in thermal stability, swelling ratio, and pH sensitivity still need to be addressed for its full-scale implementation.

## Figures and Tables

**Table 1:** Negative impacts of heavy metals and synthetic dyes on human health.

Pollutants		Potential Negative Impact on Human Health	Ref.
**Heavy metals**	Zn	Stomach cramps, dermal irritations, vomiting, nausea, and anemia	[13]
	Cu	Severe toxicological complications such as vomiting, cramps, convulsions, and even death	[14]
	Ni	Acute lung, kidney and gastrointestinal pain, pulmonary fibrosis, and skin dermatitis	[15]
	Hg	Pulmonary, kidney, and chest pain, dyspnea impairments	[16]
	Co	Paralysis, asthma, pneumonia, diarrhea, lung irritations, weight loss, vomiting, nausea, damage thyroid hormone and liver	[17,18,19]
	Cd	Renal dysfunction and even death	[13]
	Pb	Fauilure of kidney, liver, reproductive system, basic cellular processes, brain function, and even the central nervous system of the human body	[20,21,22]
	Cr	Destruction of human metabolism, food chain disruption, skin irritation, lung carcinoma	[23,24]
**Synthetic dyes**	Azo dyes	DNA destruction, carcinogenic and mutagenic, skin irritation, hypertension, tongue and larynx distress, blindness, acute tubular necrosis, gastritis, respiratory distress, liver issues, bladder cancer, neurosensory harm	[25,26,27,28,29,30]
	Reactive dyes	Respiratory diseases, asthma, coughing, wheezing, sneezing, watery eyes, itching, respiratory sensitization, poor immune system, deteriorate the water quality and damages to water bodies, ecosystem and biological cycle	[31,32,33]
	Vat dyes	Skin irritation	[33]
	Sulfur dyes	Unpleasant odor, carcinogenic, skin irritation, allergic dermatitis, mutations	[34,35]
	Disperse dyes	Bioaccumulation in nature, genotoxic in mammalian assays, mutagenic	[36]

**Table 2:** Recently published review articles on wastewater treatment using hydrogels.

Types of Hydrogel	Types of Pollutants	Major Contents	Ref.
Synthetic and natural hydrogels	Heavy metal	Selectivity, efficiency, and reusability	[57]
Synthetic and natural hydrogels	Heavy metal	Factors of adsorption and detection of metal	[58]
Synthetic and natural hydrogels	Aqueous pollutants including dye, heavy metals, and anions	Adsorption kinetics, regeneration, and reusability	[49]
Synthetic and natural hydrogels	Dye, heavy metal, radioactive materials, pesticides	Adoption properties, kinetics, isotherm, mechanism, factors, recycling, and recovery	[59]
Synthetic and natural hydrogels	Dye and metal	Synthesis, mechanism, modification of adsorbents, and kinetics	[60]
Acrylic-based hydrogels	Dye and heavy metals	Preparation and adsorption properties of different acrylic-based HG	[61]
Hybrid hydrogels	Metal, radionuclides, anions, acid phenol ammonium	Adsorption properties	[62]
Composite hydrogels	Dye	Adsorption properties of different types of composite hydrogels	[63]
Cellulose-based hydrogel	Dye and metal	Preparation, adsorption mechanisms, and factors affecting adsorption capacity	This paper

**Table 3:** Proposed removal mechanism of contaminants by CBHs.

CBHs	Synthesis Method	Pollutants	Proposed Mechanisms	Ref.
Cellulose–bentonite porous composite	Crosslinking	Azo dye	Electrostatic interaction	[144]
Carboxymethyl cellulose HG beads	Inverse suspension crosslinking	Cu^2+^, Ni^2+^, Pb^2+^	Electrostatic and coordination interactions	[105]
Chemi-mechanical pretreated cellulose-based superabsorbent HG	Modification of cellulose and acryloyl chlorides	Methylene Blue	Electrostatic interactions and H-bonding	[133]
Superadsorbent cellulose–graft–acrylic acid	Free-radical polymerization	Methylene Blue	Electrostatic interactions	[145]
Cyanoethyl cellulose	Ionic xanthate graft polymerization	Cu^2+^	Electrostatic interactions	[146]
Carboxymethyl cellulose-based magnetic superabsorbent	Simultaneous magnetic ion oxides nanoparticles and superabsorbent formation	Crystal violet	Electrostatic interactions	[147]
Cellulose–graft–polyacrylamide/hydroxyapatite composite HG	Suspension polymerization	Cu^2+^	Ion exchange	[125]
Sugar cane bagasse cellulose and gelatin-based composite HGs	Crosslinking	Cu^2+^	Electrostatic and coordination interactions	[143]
Carboxylated cellulose nanocrystal-sodium alginate HG beads	Crosslinking	Pb^2+^	Complexation and electrostatic interactions	[120]
Carboxylated cellulose nanofibrils-filled magnetic chitosan HG beads	Instantaneous gelation	Pb^2+^	Electrostatic adosption	[51]
Carboxymethyl cellulose–graft poly(acrylic acid)/monmorilonite HG composite	Graft polymerization	Pb^2+^, Zn^2+^	Ion exchange and coordination interactions	[148]
Hydroxypropyl cellulose/molybdenum disulfide composite HGs	Graft polymerization	Methylene Blue	Electrostatic interactions	[149]
Cellulose–graft–acrylic acid HGs	Grifting reaction mechanism	Cd^2+^, Ni^2+^, Pb^2+^	Electrostatic interactions and ion exchange	[124]
TEMPO-oxidized cellulose HGs	Nitroxy radical catalyzed oxidation	Zn^2+^, Fe^3+^, Cd^2+^, Cs^+^	Electrostatic interactions and ion exchange	[150]
Chitin/cellulose composite HGs	Freezing/thawing	Hg^2+^, Pb^2+^, and Cu^2+^	Electrostatic and coordination interactions	[54]
Cellulose-based bio-adsorbent	Graft copolymerization	Acid Blue, Methylene Blue	Electrostatic interactions and H-bonding	[46]
Carboxymethyl chitosan/poly (acrylonitrile) HGs	Crosslinking	Cu^2+^, Cd^2+^, and Co^2+^	Electrostatic interactions	[151]
Chitogen/Cellulose HGs	Freeze-dried	Congo Red	Electrostatic and coordination interactions	[55,152]
Carboxymethyl cellulose structured nano-adsorbent	Sol–gel method	Methyl Violet	Electrostatic and π–π interactions	[153]
Nanocomposite HG	Graft polymerization	Crystal Violet	Electrostatic interactions, H-bonding	[154]
CMC–acrylamide–graphene oxide HGs	Radical polymerization	Acid Blue 133	Electrostatic interactions	[81]
Lignocellulose-g-poly(acrylic acid)/montmorillonite 3D crosslinked polymeric netwrok HGs	Copolymerization	Methylene Blue	Electrostatic interactions	[155]
Carboxymethyl Cellulose gel	γ-irradiation	Cu^2+^	Chelation (coordination interactions)	[53]
CMC-acrylic acid adsorbent	Graft polymerization	Methyl Orange, Disperse Blue 2BLN, and Malachite Green Chloride	Electrostaic interactions	[152]
CMC/2-acrylamido-2-methyl propane sulfonic acid HGs	Copolymerization and crosslinking	Co^2+^, Cu^2+^, Mn^2+^, Fe^3+^	Electrostatic and chelating interactions	[156]

**Table 4:** Effects of initial pollutant concentration on adsorption capacities of CBHs.

Materials	Dye/Metal	Initial Concentration (mg/L)	Adsorption Capacity (mg/g)	Ref.
Cellulose-based porous adsorbent	Methylene Blue	3000 2500 1500 500	1505.2 1471.5 1175.4 237.7	[175]
Lignocellulose-based nanocomposite hydrogel	Methylene Blue	2500 2200 1800	1975 1875 1710	[155]
Carboxymethyl-based cellulose	Methyl Orange	1500 1000 500	1825 1650 950	[176]
Chitosan/cellulose hydrogels	Congo Red	500	165	[55]
Pineapple peel CBH	Methylene Blue	200	150	[55,177]
Cellulose-based adsorbent	Cd^2+^	600 1000 1600 2000	350 460 530 540	[124]
	Pb^2+^	600 1000 1600 2000	420 630 780 810	
	Ni^2+^	600 1000 1600 2000	200 320 350 360	
Carboxymethyl Cellulose	Zn^2+^	200 500	90 170	[148]
	Pb^2+^	200 500	65 110	

**Table 5:** Effects of pH on adsorption capacities of different CBHs.

Materials	Dye/Metal	pH for Max. Adsorption	Ref.
Porous cellulose-based bio-adsorbent	Methylene Blue (cationic dye)	9.0	[46]
Carboxymethyl cellulose	Cd^2+^, Pb^2+^	4.0	[180]
Cellulose–graft–acrylic acid HGs	Cd^2+^, Pb^2+^, Ni^2+^	3.0	[124]
Amide-functionalized cellulose-based porous adsorbent	Acid Black (anionic dye) Acid Red (anionic dye) Cu^2+^	2.0 2.0 7.0	[181]

**Table 6:** Effect of carboxymethyl cellulose (CMC)/2-acrylamido-2-methyl propane sulfonic acid (AMPS) composition on the adsorption of heavy metals.

AMPS Content (wt%)	Swelling (%)	Amount of Metal Ion Recovered (mg/g)			
		Co^2+^	Cu^2+^	Fe^3+^	Mn^2+^
10	581	16.3	27.4	25.3	7.1
25	617	43.1	52.7	56.8	18.6
50	690	60.6	75.3	80.4	46.8

## Supplementary Materials

The following are available online at https://www.mdpi.com/2310-2861/7/1/30/s1, Table S1: Recent works on removal of heavy metals using CBHs, Table S2: Recent works on removal of dyes using CBHs.
