# Supplementary material for: Cellulose-Based Hydrogels for Wastewater Treatment: A Concise Review
Source: Gels. 2021 Mar 18;7(1):30. doi: 10.3390/gels7010030 (PMC8005947; doi:10.3390/gels7010030)
Supplement: Supplementary file 1 [file gels-07-00030-s001.pdf]

Supplementary Materials

Cellulose-Based Hydrogels for Wastewater Treatment: A Concise Review

Table S1. Recent works on removal of heavy metals using CBHs.

Cellulose-Based Hydrogel	Hydrogel Preparation Mechanism	Metal	Adsorption Capacity, mg/g	Ref.
Cellulose beads from bleached almond shell	Dissolution- coagulation- freeze-drying method	Cu(II)	128.24	[1]
Microcrystalline Cellulose hydrogels	Self-assembly	Heavy metal	-	[2]
Microcrystalline Cellulose hydrogels	Cross-linked via freeze-drying/ physical cross-linking and hydrogen bond	Ag(I)	234.32	[3]
Microcrystalline Cellulose composite hydrogels	Chemical crosslinking	Cu(II)<br>Pb(II)	For GTD-1*<br>Cu(II)-65.1<br>Pb(II)-80.9<br>For GTD-2**<br>Cu(II)-74.2<br>Pb(II)-91.7	[4]
Cellulose filament fibers-based hydrogel	Chemical crosslinking	Cu(II)	51.3	[5]
Microcrystalline Cellulose hydrogels	Chemical crosslinking	Cr(VI)<br>Cd(II)<br>Cu(II)<br>Zn(II)<br>Pb(II)	0.0019<br>0.001995<br>0.001<br>0.003<br>0.001985	[6]
3D MnO_2 modified biochar-based porous hydrogels	Free-radical polymerization	Cd(II)<br>Pb(II)	84.76<br>70.90	[7]
Cellulosic black cumin seed powder based	Co-precipitation Graft copolymerization method	As(III)	1.0	[8]
Hydroxypropyl Cellulose Beads	Ionotropic gelation	Pb(II)	47.72	[9]
Sawdust based adsorbents	liquid phase reduction method	As(III/V)<br>Cr(III)	111.37<br>268.7	[10]
Carboxymethyl cellulose hydrogel	Free radical polymerization	Ce(III)<br>Gd(III)	205.48<br>216.73	[11]
Carboxyl methylcellulose and chitosan-derived nanostructured sorbents	Chemical cross-linking	Cd(II)<br>Cr(VI)	470.0<br>347.0	[12]
Cellulose nanocrystal composite hydrogel	Hydrothermal method	As(III)	13.866	[13]

		As(V)	15.712	
Cellulose (cotton) based adsorbent	Graft copolymerization method	Cr(VI)	490.3	[14]
Cellulose nanofibers based fluorescent hydrogel	Free-radical polymerization	Cr(VI)<br>Ba(II)<br>Pb(II)<br>Cu(II)	534.4<br>271.9<br>789.6<br>98.2	[15]
Gum Tragacanth based nanocomposite hydrogel	Microwave assisted polymerization	Hg(II)<br>Cr(VI)	666.6<br>473.9	[16]
Soybean residue-poly(acrylic acid) (SR–PAA) based hydrogel	UV radiation assisted polymerization	Cd(II)<br>Pb(II)	160.75<br>422.28	[17]
Carboxymethyl Cellulose/Poly(N-isopropylacrylamide-co-acrylic acid) hydrogel	Free radical polymerization	U(VI)	14.69	[18]
Microcrystalline cellulose hydrogel coating nanoscale Fe^0 (CH@nFe^0)	Graft copolymerization	Cr(VI)	-	[19]
Carboxymethyl cellulose based hydrogel-Poly(CMC/Methacrylic acid)	Direct radiation grafting technique	Cu(II)<br>Co(II)	21000<br>34000	[20]
Carboxymethyl cellulose based hydrogel-Poly(CMC/Acrylamide)	Direct radiation grafting technique	Cu(II)<br>Co(II)	19000<br>24000	[20]
Wheat straw cellulose-based polymeric composites	Graft copolymerization	Cu(II)	130	[21]

*Graphene oxide-triethylenetetramine-dialdehyde cellulose hydrogel in heterogeneous system.

**Graphene oxide-triethylenetetramine-dialdehyde cellulose hydrogel in homogeneous system

Table S2. Recent works on removal of dyes using CBHs.

Cellulose-Based Hydrogels	Hydrogel Preparation Mechanism	Dye	Adsorption Capacity, mg/g	Ref.
Dialdehyde carboxymethyl cellulose–gelatin based	Crosslinked via covalent bond leading Schiff base reaction	Rhodamine B<br>Methyl Violet	763.4<br>584.7	[22]
Cellulose hydrogels physically crosslinked by glycine	Self-assembly	Dye adsorption	-	
Microcrystalline cellulose-based hydrogel	Crosslinked via hydrogen bond, physical crosslinking	Methylene Blue	13381.62	[3]
Sugar-beet pulp-based hydrogel	Instantaneous gelation	Methylene Blue	1428.6	[23]
Trimethyl ammonium grafted cellulose foams; cationic cellulose foam	Grafting and chemical crosslinking	Anionic Dye Eosin Y	364.22	[24]
Tunicate cellulose hydrogels	Dissolution-regeneration process	Methylene Blue	-	[25]
Amide-functionalized cellulose-based	Chemical crosslinking	Acid Black 1<br>Acid Red 18	751.8<br>417.9	[5]
Cellulose/biopolymer/Fe_3O_4 hydrogel microbeads	Sol–gel transition	Crystal violet<br>Methyl Orange	62<br>14	[26]
Carboxylated cellulose nanocrystal– MnO_2 beads	Physical crosslinking	Methylene Blue	136.7	[27]
Cellulosic black cumin seed powder based	Graft copolymerization method	Methylene Blue	10.0	[8]
Cellulose (cotton linters, DP 500) based hydrogels	Coagulation and regeneration	Methylene Blue	-	[28]
Nanocrystalline cellulose hydrogels	Chemical crosslinking	Methyl Orange	193	[29]
Thiol-modified Carboxymethyl cellulose nanocrystal hydrogel	Chemical crosslinking	Methylene Blue	756	[30]
Lemongrass leaf based novel cellulose biosorbent	-	Crystal Violet	36.10	[31]
Bagasse based nanocomposite hydrogel	-	Drimarine Yellow HF	-	[32]
Carboxymethyl cellulose-based hydrogel	Graft copolymerization method	Acid Red 73	-	[33]
Carboxymethyl cellulose-g-polyacrylamide/montmorillonite nanocomposite hydrogel	Free radical polymerization	Malachite green	172.4	[34]
Carboxymethyl cellulose -polyaniline hydrogel	Free radical polymerization	Methylene Blue<br>Rohdamin B<br>Methyl Orange	12.2<br>8.79<br>6.11	[35]
Cellulose nanowhiskers (cotton powder) based hybrid hydrogel	Sol–gel transition	Methylene Blue	-	[36]
Carboxymethyl cellulose based hydrogel-Poly(CMC/Methacrylic acid)	Direct radiation grafting technique	Acid blue<br>Methyl green	1800<br>2400	[20]
Carboxymethyl cellulose based hydrogel-Poly(CMC/Acrylamide)	Direct radiation grafting technique	Acid blue<br>Methyl green	1600<br>2800	[20]

References

1. Maaloul, N.; Oulego, P.; Rendueles, M.; Ghorbal, A.; Díaz, M. Synthesis and characterization of eco-friendly cellulose beads for copper (II) removal from aqueous solutions. *Env. Sci. Pollut. R.* **2019**, *27*, 23447–23463, doi:10.1007/s11356-018-3812-2.
2. Palantöken, S.; Bethke, K.; Zivanovic, V.; Kalinka, G.; Kneipp, J.; Rademann, K. Cellulose hydrogels physically crosslinked by glycine: Synthesis, characterization, thermal and mechanical properties. *J. Appl. Polym. Sci.* **2019**, *137*, 48380, doi:10.1002/app.48380.
3. Yang, K.; Li, X.; Cui, J.; Zhang, M.; Wang, Y.; Lou, Z.; Shan, W.; Xiong, Y. Facile synthesis of novel porous graphene-like carbon hydrogel for highly efficient recovery of precious metal and removal of organic dye. *Appl. Surf. Sci.* **2020**, *528*, 146928, doi:10.1016/j.apsusc.2020.146928.
4. Wang, Z.; Yao, M.; Wang, X.; Li, S.; Liu, Y.; Yang, G. Influence of reaction media on synthesis of dialdehyde cellulose/GO composites and their adsorption performances on heavy metals. *Carbohyd. Polym.* **2020**, *232*, 115781, doi:10.1016/j.carbpol.2019.115781.
5. Liu, J.; Chen, T.-W.; Yang, Y.-L.; Bai, Z.-C.; Xia, L.-R.; Wang, M.; Lv, X.-L.; Li, L. Removal of heavy metal ions and anionic dyes from aqueous solutions using amide-functionalized cellulose-based adsorbents. *Carbohyd. Polym.* **2020**, *230*, 115619, doi:https://doi.org/10.1016/j.carbpol.2019.115619.
6. Zhou, H.; Zhu, H.; Xue, F.; He, H.; Wang, S. Cellulose-based amphoteric adsorbent for the complete removal of low-level heavy metal ions via a specialization and cooperation mechanism. *Chem. Eng. J.* **2020**, *385*, 123879, doi:10.1016/j.cej.2019.123879.
7. Wu, Z.; Chen, X.; Yuan, B.; Fu, M.-L. A facile foaming-polymerization strategy to prepare 3D MnO2 modified biochar-based porous hydrogels for efficient removal of Cd(II) and Pb(II). *Chemosphere* **2020**, *239*, 124745, doi:10.1016/j.chemosphere.2019.124745.
8. Tara, N.; Siddiqui, S.I.; Nirala, R.K.; Abdulla, N.K.; Chaudhry, S.A. Synthesis of antibacterial, antioxidant and magnetic Nigella sativa-graphene oxide based nanocomposite BC-GO@Fe3O4 for water treatment. *Colloid Interface Sci. Commun.* **2020**, *37*, 100281, doi:10.1016/j.colcom.2020.100281.
9. Soleh Setiyawan, A.; Guerrero, R.; Acibar, C.; Alarde, C.M.; Maslog, J.; Pacilan, C.J.; Dwi Ariesyady, H.; Nastiti, A.; Roosmini, D.; Sonny Abfertiawan, M. Evaluation of Pb (II) Removal from Water Using Sodium Alginate/Hydroxypropyl Cellulose Beads. *E3S Web Conf.* **2020**, *148*, 02002, doi:10.1051/e3sconf/202014802002.
10. Li, X.; Zhang, J.; Xie, H.; Pan, Y.; Liu, J.; Huang, Z.; Long, X.; Xiao, H. Cellulose-based adsorbents loaded with zero-valent iron for removal of metal ions from contaminated water. *Env. Sci. Pollut. R.* **2020**, *27*, 33234–33247, doi:10.1007/s11356-020-09390-z.
11. Wang, F.; Zhu, Y.; Wang, A. Preparation of Carboxymethyl Cellulose-g- Poly(acrylamide)/Attapulgite Porous Monolith With an Eco-Friendly Pickering-MIPE Template for Ce(III) and Gd(III) Adsorption. *Front. Chem.* **2020**, *8*, doi:10.3389/fchem.2020.00398.
12. Li, S.-S.; Wang, X.-L.; An, Q.-D.; Xiao, Z.-Y.; Zhai, S.-R.; Cui, L.; Li, Z.-C. Upon designing carboxyl methylcellulose and chitosan-derived nanostructured sorbents for efficient removal of Cd(II) and Cr(VI) from water. *Int. J. Biol. Macromol.* **2020**, *143*, 640–650, doi:10.1016/j.ijbiomac.2019.12.053.
13. Dong, F.; Xu, X.; Shaghaleh, H.; Guo, J.; Guo, L.; Qian, Y.; Liu, H.; Wang, S. Factors influencing the morphology and adsorption performance of cellulose nanocrystal/iron oxide nanorod composites for the removal of arsenic during water treatment. *Int. J. Biol. Macromol.* **2020**, *156*, 1418–1424, doi:10.1016/j.ijbiomac.2019.11.182.
14. Liang, X.; Liang, B.; Wei, J.; Zhong, S.; Zhang, R.; Yin, Y.; Zhang, Y.; Hu, H.; Huang, Z. A cellulose-based adsorbent with pendant groups of quaternary ammonium and amino for enhanced capture of aqueous Cr(VI). *Int. J. Biol. Macromol.* **2020**, *148*, 802–810, doi:10.1016/j.ijbiomac.2020.01.184.
15. Luo, Q.; Yuan, H.; Zhang, M.; Jiang, P.; Liu, M.; Xu, D.; Guo, X.; Wu, Y. A 3D porous fluorescent hydrogel based on amino-modified carbon dots with excellent sorption and sensing abilities for environmentally hazardous Cr(VI). *J. Hazard. Mater.* **2021**, *401*, 123432, doi:10.1016/j.jhazmat.2020.123432.
16. Sharma, B.; Thakur, S.; Trache, D.; Yazdani Nezhad, H.; Thakur, V.K. Microwave-Assisted Rapid Synthesis of Reduced Graphene Oxide-Based Gum Tragacanth Hydrogel Nanocomposite for Heavy Metal Ions Adsorption. *Nanomaterials* **2020**, *10*, 1616, doi:10.3390/nano10081616.
17. Zhang, M.; Yin, Q.; Ji, X.; Wang, F.; Gao, X.; Zhao, M. High and fast adsorption of Cd(II) and Pb(II) ions from aqueous solutions by a waste biomass based hydrogel. *Sci. Rep.* **2020**, *10*, doi:10.1038/s41598-020-60160-w.
18. Tan, J.; Xie, S.; Wang, G.; Yu, C.W.; Zeng, T.; Cai, P.; Huang, H. Fabrication and Optimization of the Thermo-Sensitive Hydrogel Carboxymethyl Cellulose/Poly(N-isopropylacrylamide-co-acrylic acid) for U(VI) Removal from Aqueous Solution. *Polymers* **2020**, *12*, 151, doi:10.3390/polym12010151.
19. Wang, Y.; Yu, L.; Wang, R.; Wang, Y.; Zhang, X. A novel cellulose hydrogel coating with nanoscale Fe0 for Cr(VI) adsorption and reduction. *Sci. Total Environ.* **2020**, *726*, 138625, doi:10.1016/j.scitotenv.2020.138625.
20. Abdel Ghaffar, A.M.; El-Arnaouty, M.B.; Abdel Baky, A.A.; Shama, S.A. Radiation-induced grafting of acrylamide and methacrylic acid individually onto carboxymethyl cellulose for removal of hazardous water pollutants. *Des. Monomers Polym.* **2016**, *19*, 706–718, doi:10.1080/15685551.2016.1209630.

21. Kong, W.; Li, Q.; Li, X.; Su, Y.; Yue, Q.; Zhou, W.; Gao, B. Removal of copper ions from aqueous solutions by adsorption onto wheat straw cellulose-based polymeric composites. *J. Appl. Polym. Sci.* **2018**, *135*, 46680, doi:10.1002/app.46680.
22. Sethi, S.; Kaith, B.S.; Kaur, M.; Sharma, N.; Khullar, S. A hydrogel based on dialdehyde carboxymethyl cellulose–gelatin and its utilization as a bio adsorbent. *J. Chem. Sci.* **2019**, *132*, doi:10.1007/s12039-019-1700-z.
23. Moharrami, P.; Motamedi, E. Application of cellulose nanocrystals prepared from agricultural wastes for synthesis of starch-based hydrogel nanocomposites: Efficient and selective nanoadsorbent for removal of cationic dyes from water. *Bioresour. Technol.* **2020**, *313*, 123661, doi:10.1016/j.biortech.2020.123661.
24. Feng, C.; Ren, P.; Huo, M.; Dai, Z.; Liang, D.; Jin, Y.; Ren, F. Facile synthesis of trimethylammonium grafted cellulose foams with high capacity for selective adsorption of anionic dyes from water. *Carbohyd. Polym.* **2020**, *241*, 116369, doi:10.1016/j.carbpol.2020.116369.
25. Wang, J.; Li, X.; Cheng, Q.; Lv, F.; Chang, C.; Zhang, L. Construction of β-FeOOH@tunicate cellulose nanocomposite hydrogels and their highly efficient photocatalytic properties. *Carbohyd. Polym.* **2020**, *229*, 115470, doi:10.1016/j.carbpol.2019.115470.
26. Park, S.; Oh, Y.; Yun, J.; Yoo, E.; Jung, D.; Oh, K.K.; Lee, S.H. Cellulose/biopolymer/Fe3O4 hydrogel microbeads for dye and protein adsorption. *Cellulose* **2020**, *27*, 2757–2773, doi:10.1007/s10570-020-02974-5.
27. Diao, H.; Zhang, Z.; Liu, Y.; Song, Z.; Zhou, L.; Duan, Y.; Zhang, J. Facile fabrication of carboxylated cellulose nanocrystal–MnO2 beads for high-efficiency removal of methylene blue. *Cellulose* **2020**, *27*, 7053–7066, doi:10.1007/s10570-020-03260-0.
28. Chen, Y.; Xiang, Z.; Wang, D.; Kang, J.; Qi, H. Effective photocatalytic degradation and physical adsorption of methylene blue using cellulose/GO/TiO2 hydrogels. *RSC Adv.* **2020**, *10*, 23936–23943, doi:10.1039/d0ra04509h.
29. Safavi-Mirmahalleh, S.-A.; Salami-Kalajahi, M.; Roghani-Mamaqani, H. Adsorption kinetics of methyl orange from water by pH-sensitive poly(2-(dimethylamino)ethyl methacrylate)/nanocrystalline cellulose hydrogels. *Env. Sci Pollut R* **2020**, *27*, 28091–28103, doi:10.1007/s11356-020-09127-y.
30. Li, Y.; Hou, X.; Pan, Y.; Wang, L.; Xiao, H. Redox-responsive carboxymethyl cellulose hydrogel for adsorption and controlled release of dye. *Eur. Polym. J.* **2020**, *123*, 109447, doi:10.1016/j.eurpolymj.2019.109447.
31. Putri, K.N.A.; Keereerak, A.; Chinpa, W. Novel cellulose-based biosorbent from lemongrass leaf combined with cellulose acetate for adsorption of crystal violet. *Int. J. Biol. Macromol.* **2020**, *156*, 762–772, doi:10.1016/j.ijbiomac.2020.04.100.
32. Kausar, A.; Shahzad, R.; Iqbal, J.; Muhammad, N.; Ibrahim, S.M.; Iqbal, M. Development of new organic-inorganic, hybrid bionanocomposite from cellulose and clay for enhanced removal of Drimarine Yellow HF-3GL dye. *Int. J. Biol. Macromol.* **2020**, *149*, 1059–1071, doi:10.1016/j.ijbiomac.2020.02.012.
33. Zhou, Y.; Shen, J.; Bai, Y.; Li, T.; Xue, G. Enhanced degradation of Acid Red 73 by using cellulose-based hydrogel coated Fe3O4 nanocomposite as a Fenton-like catalyst. *Int. J. Biol. Macromol.* **2020**, *152*, 242–249, doi:10.1016/j.ijbiomac.2020.02.200.
34. Peighambardoust, S.J.; Aghamohammadi-Bavil, O.; Foroutan, R.; Arsalani, N. Removal of malachite green using carboxymethyl cellulose-g-polyacrylamide/montmorillonite nanocomposite hydrogel. *Int. J. Biol. Macromol.* **2020**, *159*, 1122–1131, doi:10.1016/j.ijbiomac.2020.05.093.
35. Bagheri, N.; Mansour Lakouraj, M.; Hasantabar, V.; Mohseni, M. Biodegradable macro-porous CMC-polyaniline hydrogel: Synthesis, characterization and study of microbial elimination and sorption capacity of dyes from waste water. *J. Hazard. Mater.* **2021**, *403*, 123631, doi:10.1016/j.jhazmat.2020.123631.
36. Morshed, M.N.; Al Azad, S.; Deb, H.; Shaun, B.B.; Shen, X.L. Titania-loaded cellulose-based functional hybrid nanomaterial for photocatalytic degradation of toxic aromatic dye in water. *J. Water Process Eng.* **2020**, *33*, 101062, doi:10.1016/j.jwpe.2019.101062.
